# Starch-Derived Bioplastics: Pioneering Sustainable Solutions for Industrial Use

**DOI:** 10.3390/ma18081762

**Published:** 2025-04-11

**Authors:** Mahmoud Omar Sobeih, Shadi Sawalha, Rinad Hamed, Fathilah Ali, Minsoo P. Kim

**Affiliations:** 1 Department of Chemical Engineering and Sustainability, Kulliyyah of Engineering, International Islamic University Malaysia, Kuala Lumpur 53100, Malaysia; mahmoudsobeih30@gmail.com; 2 Department of Chemical Engineering, An Najah National University, Nablus P.O. Box 7, Palestinian Territory; sh.sawalha@najah.edu; 3 Department of Chemistry, Faculty of Science, An-Najah National University, Nablus P.O. Box 7, Palestinian Territory; rinad.hamed@najah.edu; 4 Department of Chemical Engineering, Sunchon National University, Suncheon 57922, Republic of Korea

**Keywords:** starch-based bioplastic, essential oils, natural fillers, nanoparticles, properties, applications, biodegradability, sustainability

## Abstract

The use of plastics has increased due to the increase in population and applications in various industries. However, fossil fuel-based plastics have caused environmental issues and health hazards due to their non-degradable behavior. To resolve the on-going crisis of these non-degradable polymers, biopolymers have been considered as potential substitutes. Starch is being researched as a polymer matrix to develop bioplastics. Starch is abundant, but due to its poor water barrier and mechanical properties, other materials need to be incorporated in the matrix to improve the material properties. Natural fillers, plasticizers, essential oils, nanoparticles, or polymer blends are materials that can be used in starch-based bioplastics. Adding these materials enhances the mechanical and barrier properties. This review summarizes the recent developments in starch-based bioplastics and biocomposites and discusses the types of starch used, fillers, essential oils, and nanoparticles, explaining how they improve the mechanical, barrier, antibacterial, and biodegradability properties. Furthermore, many of the research products show potential to be used in industrial applications like packaging and agriculture. This review also discusses the potential of starch bioplastics in industrial applications like packaging, automotive applications, biomedical applications, electronics, construction, textiles, and consumer goods. This review also discusses the environmental impact of starch-derived bioplastic products, the life cycle, biodegradation, and recycling process. The circular economy of bioplastics, the economic feasibility of large-scale products, and regulation were also discussed, along with their challenges and the future perspectives of starch-based bioplastics.

## 1. Introduction

Plastics are used in a wide range of applications, such as domestic, industrial, and commercial applications, but as the world’s population continues to increase, the demand for plastic increases. Plastics are inexpensive, light-weight, and durable; thus, global plastic production is expected to reach a projected value of 1.1 billion metric tons by 2050 [1]. However, plastic causes pollution, impacting ecosystems due to its durability, which means it decomposes over a long period [2,3]. Research shows that 220 million tons of plastic was generated, averaging 28 kg per person worldwide, and it is predicted that 420 million tons will be generated in 2040 [4]. Plastic pollution is a global environmental challenge due to plastics ending up in landfills and incineration plants or being improperly disposed of. This widespread use results in plastics polluting terrestrial and aquatic systems and causes the spread of microplastics, which have health effects [5]. Also, greenhouse gas emissions occur at every stage of the plastic life cycle, which affect the environment through phenomena such as global warming [6]. To overcome these challenges, there is a need to produce plastics from natural renewable sources.

**Figure 1:**
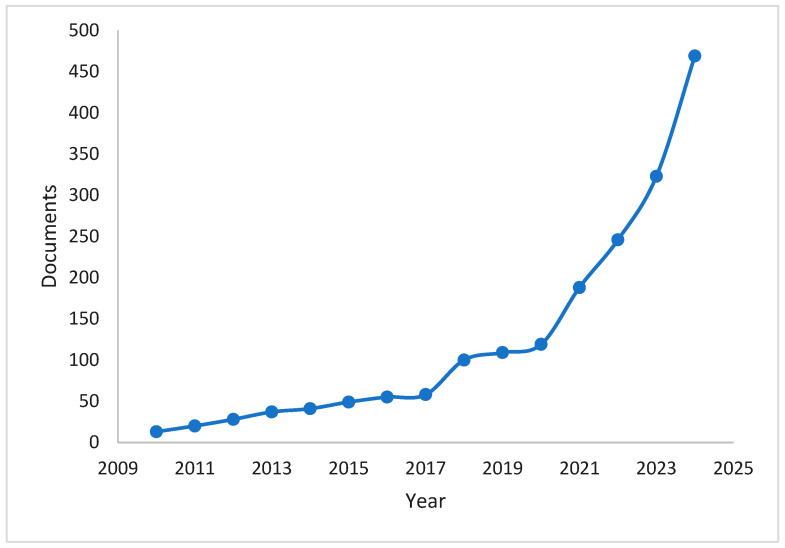
Articles published with the following keywords: starch bioplastics and biocomposites (source: Scopus).

Bioplastics are derived from renewable biomass such as plants and plant products, which easily break down without harming the environment. They are economically friendly, renewable, and non-toxic due to their botanical sources such as starch, cellulose, proteins, lipids, and more. Also, they reduce non-biodegradable waste and save energy during production [7]. Bioplastics have the potential to significantly reduce plastic pollution in the environment, and research shows that bioplastics can reduce carbon dioxide emissions by 30–70% [8]. The most used and promising bioplastics are based on starch due to its abundance, low cost, renewability, and sustainability at the industrial scale. However, starch has the disadvantages of a poor water barrier and mechanical properties, and to overcome these issues, additives like natural fillers, essential oils, nanoparticles, and polymer blends are added into the composite matrix. These additives reinforce the polymer matrix, enhancing mechanical and barrier properties, and promote biodegradability. For the past 10 years, many scientists and researchers have developed starch-based biocomposites and enhanced their properties [9,10,11], as shown in Figure 1. Several review articles have discussed different aspects of starch-based bioplastics, including the works of Jayarathna et al. [12] and Rahardiyan et al. [13]. This review aims to provide a more focused and updated analysis of recent advancements in the incorporation of natural fillers, essential oils, nanoparticles, and polymer blends in starch-based bioplastics. It will particularly emphasize their effects on mechanical, barrier, and antibacterial properties, highlighting their potential for industrial applications. Furthermore, this review explores current industrial applications—including packaging, agriculture, textiles, automotive applications, consumer goods, and construction—while addressing the environmental and economic impacts of bioplastics. Finally, the existing challenges in the field are discussed, and future directions for further innovation and commercialization are proposed.

## 2. Starch-Based Bioplastics

**Figure 2:**
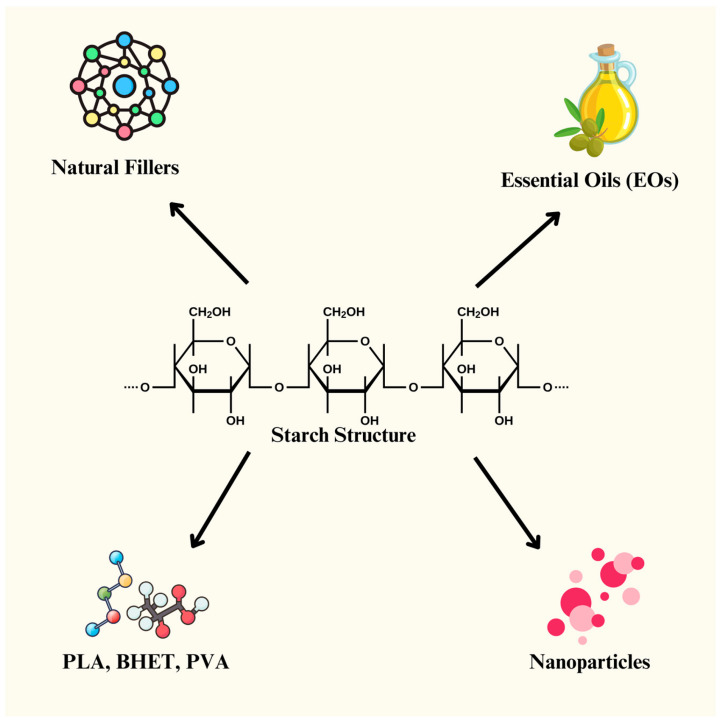
Molecular structures of starch and reinforced fillers and polymer blends.

Starch is a biopolymer consisting of d-glucose subunits connected by glycosidic bonds containing varying amounts of amylopectin (80–90%) and amylose (10–20%), where amylose is a linear polysaccharide with α-(1-4)-linked d-glucose units and amylopectin is a highly branched molecule with α-(1-6)-linked branches [14]. The higher proportion of amylopectin increases the crystallinity of starch, whereas amylose provides better tensile strength, a lower elongation at break, and a higher Young’s modulus [15]. From these properties, starch has great potential to be used to develop bioplastics due to its biodegradability, renewability, and availability in large quantities, and currently, 50% of developed bioplastics are starch-based; thus, it is predicted to be the dominant material source in the industry by 2030 [16,17]. Many previous studies have produced bioplastics from natural starch sources such as banana peel [18], corn [19], rice [20], cassava [21], tapioca [22], sago [23], and potato starch [24]. Over time, many researchers have found innovative ways to improve the properties by adding natural fillers, essential oils, nanoparticles, and polymer blends (PLA, BHET, and PVA), as is shown in Figure 2. This section discusses the recent work performed in developing starch-based bioplastics.

### 2.1. Natural Fillers

Natural fillers are bio-based materials that are added into a polymer, which improve the mechanical, thermal, and barrier properties. Also, they have great advantages of easy availability, a simple manufacturing process, renewability, and less energy consumption. The most widely used natural fillers are natural fibers from plants because of their abundance, availability, and low cost. Plant fibers include kenaf, jute, sugarcane, bagasse, oil palm fruit, husk, rice, straw, and more. A higher cellulose content allows for the fabrication of composites with high strength [25,26]. This section discusses the works performed regarding different types of natural fillers and enhancement properties, stressing the importance of adding them into the polymer matrix. The findings are also summarized in Table 1.

#### 2.1.1. Plant Fillers

Bioplastics have been studied with a focus on packaging applications. A heat-sealable bioplastics film was developed from locust bean and potato byproducts. The samples were prepared from a blend of locust bean milling dust (5% *w*/*v*) and starch (2% or 4% *w*/*v*) dispersed in distilled water with glycerol. This work analyzed chromatic, morphological, and mechanical properties and carried out gas barrier testing. After that, cheese and oat cookies were packaged and stored at 4 °C. The tensile strength (TS) and elongation at break (EAB) were 4.3 MPa and 64.5%. The bioplastic prepared from locust bean milling dust (LBMD) and starch developed transparent and yellow films with higher rigidity, hydrophobicity, and gas barrier properties. The quality of the cheese was protected for 14 days of storage, but after that, oxidative reactions occurred through odor and flavor. The oat cookies were preserved longer at 21 days as these bioplastic films prevented the oxidative rancidity of fatty cookies. This LBMD/starch bioplastic was proven to preserve fatty foodstuffs [27].

A viable fiber that can be made from the entire plant is kenaf because of its low cost; it enhances mechanical properties and has diverse applications in many climates. Hazrol et al. [28] developed a corn starch biocomposite incorporating kenaf fiber at different concentrations (0, 2, 4, 6, and 8 wt%. of corn starch). The optimal biocomposite was that with 6 wt% with a high TS of 17.74 MPa and an EAB of 48.79%; it absorbed less water and decreased the moisture content (5.99%). The incorporation of kenaf fiber enhances the mechanical properties, improves barrier properties, and can solve the fragility and brittleness of starch films.

Majamo et al. [29] extracted anchote starch and reinforced enset fiber as it has significant chemical and physical qualities that make it a valuable raw material. The bioplastic film was prepared using 100 mL of distilled water and 5 g of extracted starch with enset fiber mixed in varying ratios (0%, 4%, 8%, 12%, 16%, and 20% *w*/*w* starch), hydrochloric acid (HCl), and glycerol. The mechanical properties were tested using ASTM D3039 [30], where 100 mm by 10 mm film specimens were created. The tensile strength increased with an increased fiber loading until reaching 8% with a maximum tensile strength of 8.34 MPa. The increase in fiber decreased the elongation at break, where at a 16% fiber loading, the elongation at break was 16.86%, and the fiber loading helped decrease the moisture content and water solubility. This work suggests that the bioplastic with 8% fiber loading qualifies as a packaging material due to its high transparency, flexibility, and stability.

Chaffa el al. [31] used false banana fiber (*Ensete ventricosum*) to develop a starch-based bioplastic from rotten potato peels. The bioplastic was developed by mixing potato peel starch with optimized concentrations of HCl and glycerol and a drying temperature 48 °C with different concentrations of false banana fiber (2, 4, 6, 8, and 10% *w*/*w* of starch). The optimized concentrations of HCl (3.5 mL) and glycerol (3 mL) and the drying temperature (48 °C) were achieved using a three-level, three-factor Box–Behnken experimental design and provided a TS of 6.449 and EAB of 19.87%. As the fiber loading increased, the TS increased until reaching 6% *w*/*w* (8.878 MPa) and then decreased, the EAB decreased (from 19.87% to 16.96%), water absorption decreased (59.94%), and biodegradability showed a 83.92% weight loss. This work showed that adding fibers into the matrix enhances the mechanical properties and improves the barrier properties; thus, the product can be used for packaging.

Santana et al. [32] used brown seaweed (*Rugulopteryx okamurae*), from the bay of Algeciras, to develop a casava starch-based biocomposite with different ratios of seaweed and cassava starch (100/0, 70/30, 50/50, and 30/70). The TS and EAB increased when cassava starch increased. The TS increased from 0.3 to 0.8 MPa, and the EAB from 1.98 to 2.52%, and the product showed thermal stability from degradation at 200–300 °C.

Apple pomace is the solid residue obtained after the processing of apples containing cellulose, lignin, starch, pectin, and small quantities of protein, showing potential as an additive. Ekielski et al. [33] used design of experiments to develop biocomposites with different weight percentages of apple pomace powder and potato starch and different moisture percentages. This included 14 samples with different apple pomace (60–100 wt%), potato starch (0–40 wt%), and moisture (10–14%) contents to determine the bonding strength, Young’s modulus, and wetting angle. The strongest materials were the biocomposites with the highest amount of starch with moisture contents of 10% and 14%, and the one with the 10% moisture content showed better temperature resistance. The results show that combining apple pomace and potato starch has the potential to produce biodegradable materials like plates and cups.

Coffee grounds have fertilizing functions, which give them the advantage of being a filler for biocomposites. They contain high amounts of polysaccharides, sugars, oils, antioxidants, phenolic compounds, and other high-value compounds like iron and zinc. Zdanowicz et al. [34] developed corn starch thermoplastic films with choline chloride:urea (CCU) and betaine:urea (BU) mixtures at a molar ratio of 1:5 with 20 pph of coffee ground filler. The addition of coffee ground increased the TS for the CCU (1.75 to 3.4 MPa) and BU (3.3 to 4.4 MPa) samples and decreased the EAB for the CCU (79 to 31%) and BU (52 to 30%) samples. The coffee grounds increased the moisture content for both CCU (13.2 to 14.7%) and BU (14.2 to 15.5%) but decreased the swelling degree (CCU: 375 to 240%; BU: 349 to 277%). The biodegradation results showed that all samples degraded within 70 days; however, the BU sample degraded slower. Using coffee grounds as a filler enhanced the mechanical properties and improved the barrier properties, showing that they can be applied as fertilizer carriers in agriculture bioplastics.

A natural filler and reinforcement agent, banana pseudostem, has been used in developing a cassava starch biocomposite. Dilkushi et al. [35] used cassava starch, glycerol, poly(vinyl alcohol), and banana pseudostem powder to develop a biocomposite with different compositions of PVA (10, 15, 20, 25, 30, 35, and 40%) and pseudostem powder from sour plantain and ash plantain (10, 15, 20, 25, 30, 35, and 40%), producing a total of 14 films. The addition of PVA uniformly improved the texture as compared to samples without PVA. The optimal composition was the biocomposite with 30% pseudostem powder, resulting in exceptional mechanical properties (TS of 2.5 MPa and EAB of 11%), physiochemical properties (water absorption 50%), and thermal stability with an onset temperature of 120 °C, giving it great potential to be used for food packaging.

Aaliya et al. [36] used five plant sources of mucilage to develop a bioplastic. Talipot starch was mixed with distilled water, glycerol, and 5% (*w*/*v*) of each of the following mucilage solutions: shoeblack leaves, okra, basil seeds, fenugreek seeds, and flax seeds. A bioplastic film without any mucilage was used as the control sample. The basil seed biocomposite had the lowest moisture content (7.2%), a low EAB (79%), and the highest water solubility (19%), water vapor permeability, and tensile strength (8 MPa). The decomposition temperatures were significantly higher, showing increased thermal stability. The biocomposite without mucilage completely degraded after the 10th day. The basil seed biocomposite also had the highest degradation stability and decomposed in 15 days with an 89% weight loss. In general, all developed mucilage biocomposite films had improved barrier properties, mechanical strength, and biodegradation stability, which makes them promising innovative sustainable packaging materials that can enhance the protection of food products.

Gum Arabic is a natural gum derived from acacia trees and can be used as a thickening agent with its amphiphilic nature and exceptional film-forming properties. Oluba et al. [37] developed a ginger starch–gum Arabic biocomposite in which 29 mL of gum Arabic solution (15% *w*/*v*) and 1 mL of ginger starch solution (5% *w*/*v*), and 28 mL of gum Arabic solution (15% *w*/*v*) plus 2 mL ginger starch solution (5% *w*/*v*) with distilled water and glycerol, were used for physiochemical and mechanical testing and coating applications. The water solubility decreased by 13% due to a higher starch content than the 1 mL solution. The biocomposite with a ratio of 28:2 had the highest moisture content (9.5%), thickness (0.25 mm), TS (15.3 MPa), and EAB (38.6%) and was thermally stable. Four coating solutions were prepared with distilled water (control), gum Arabic solution, ginger starch solution, gum Arabic–ginger starch composite at a ratio of 29:1, and gum Arabic–ginger starch composite at a ratio of 28:2 to coat tomatoes to analyze the quality and shelf life of the tomatoes for 20 days. Using gum Arabic–ginger starch biocomposites as a coating for tomatoes led to a 10.3% reduction in weight loss, demonstrating the potential of the biocomposite as a sustainable packaging material for the post-harvest storage of tomatoes.

Another natural filler is olive pit powder (OPP) composed of cellulose (20.9%), hemicellulose (26%), and lignin (35.6%), which are major solid wastes from olive oil extraction. Lounis et al. [38] developed a bioplastic with corn starch, glycerol, acetic acid, and different concentrations of OPP (0, 10, 20, 30, 50, and 70% *w*/*w* starch), as well as two other films with antibacterial agents such as zinc oxide (ZO) and oregano essential oil with a 20% OPP concentration. There was an increase in the moisture content from 9.2% to 14.7% when OPP was added, and it decreased to 11.56% with a higher OPP concentration, and there was a decrease in the swelling degree index from 121.9 to 79.1%, though improved water barrier abilities were seen. Mechanical testing was not performed in this work, but biodegradability was observed in 14 days. The 70% OPP concentration had the highest weight loss of 91%. In terms of antibacterial assay, the film with oregano essential oil–OPP 20% had the highest antibacterial effects against *S. aureus* (35 mm), *E. coli* (32 mm), and *Salmonella* (30 mm), and ZnO-OPP 20% also had antibacterial effects against *S. aureus* (19 mm), *E. coli* (18 mm), and *Salmonella* (18 mm), just like the control film with OPP 20%. Biodegradable packaging material made from corn starch and OPP can be a great alternative as it enhances water resistance and antimicrobial properties.

Torres-Vargas et al. [39] developed a corn starch-based biocomposite with different concentrations of natural filler cellulose nanocrystals from corn husk (CCNC) (2, 4, 6, and 8% *w*/*w*) and tested it through packaging preservation with cherry tomatoes. The addition of CCNC increased the TS by 5.67 MPa and decreased the EAB by 44% due to the formation of a rigid straight chain gel matrix through a strong intermolecular interaction between the polymers. Regarding the cherry tomatoes, weight loss was calculated for a storage period of 9 days. After nine days, the cherry tomatoes lost moisture with surface mold from biocomposites with 2 and 4 wt%., and the 6 and 8 wt%. biocomposites kept the tomatoes in good condition due to its good vapor permeability. CCNCs are promising reinforcement materials due to their high mechanical properties and thermal stability, and they showed potential to extend the shelf life of cherry tomatoes.

Charles et al. [40] recently developed a biocomposite using arrowroot starch and sodium alginate with coconut jelly powder as a filler (1, 2, and 3% *v*/*v*) to test mechanical and physiochemical properties and then coated the solution over cherry tomatoes to test weight loss. The addition of coconut jelly powder increased the TS from 1.84 to 9.35 MPa and decreased the EAB from 91.33 to 32.8%. Regarding the physicochemical properties, the moisture content (33.44 to 18.92%) and water solubility (36.79 to 25.46%) decreased due to the hydrophilic nature of arrowroot starch. The addition of coconut jelly powder improved the quality of the cherry tomatoes with a weight loss of 8.19%. All the films exhibited soil biodegradation in less than 28 days, and using coconut jelly powder as a filler enhanced the mechanical and thermal properties, showing that it has potential to be used as packaging material to promote a longer shelf life.

Aloe vera gel contains polysaccharides, which promote antibacterial activity and mechanical properties. Guno et al. [41] used the Box–Behnken Response Surface Methodology to develop and optimize biocomposites films with three independent variables, namely taro starch (25 to 35%), glycerol (0 to 2%), and Aloe vera gel (30 to 150%), and response variables such as the water vapor transmission rate, tensile strength, and thickness. The optimal values of the biocomposite film were 5.56% taro starch, 25% glycerol, and 49.25% Aloe vera gel, giving the lowest water vapor transmission rate (0.00163 g/m^2^t), the highest TS (3.26 MPa), and a thickness of 0.14 mm. The outcome of this work showed the ability of the taro starch biocomposite film’s potential for usage as a sustainable and environmentally friendly packaging material.

Another recent filler that brought interest was the use of spider web due to its fracture toughness and high elongation at break and tensile strength. Kedir et al. [42] developed a biocomposite with spider web and Rosmarinus officinalis essential oil (ROEO). The films were prepared with chitosan only, potato starch/chitosan, chitosan/potato starch/spider web (0.75 *w*/*w* of chitosan starch) and chitosan/potato starch/spider web/ROEO (1, 5, and 10%) and then characterized. After testing, tomatoes were dipped in the coating solutions of the biocomposites and then dried and stored at room temperature to calculate weight loss. The spider web reinforcement enhanced the mechanical properties, namely the TS (27.5 MPa) and EAB (22.2%), and the addition of ROEO increased the TS (30.9 MPa) and EAB (19%). These fillers lowered the moisture content (46.9 to 35.91%) and water solubility (69.09 to 40.21%) when compared to the films without the fillers. In terms of antimicrobial properties, the ROEO increased the antimicrobial activity against *S. aureus*, and the minimum weight loss in tomatoes was recorded with the ROEO film (6.8%). This modified biocomposite was proven to have the potential to be used in bioactive food packaging applications due to the reduction in microbial growth and biodegradation after 60 days, showing that it can improve shelf life in coating and packaging.

#### 2.1.2. Natural Mineral Fillers

Clay minerals are another type of natural fillers, and they have been used in developing bioplastics. These include halloysite nanotube (HNT), zeolite, hectorite, montmorillonite (MMT), and bentonite, and they have the potential to improve properties and characteristics like uniform dispersion and aspect ratio.

Bentonite comprises montmorillonite belonging to the 2:1 phyllosilicates group of clay, which has been used as a filler in the polymer matrix. Behera et al. [43] developed a bioplastic with yam starch, glycerol, and different proportions of bentonite (0.5, 1, and 1.5% *w*/*w*). It was observed that the TS increased as the concentration of bentonite increased, with an optimal TS (4.063 MPa) at 1.5% *w*/*w*. Also, the 1.5% bentonite bioplastic had low water absorption and exhibited the highest soil degradation rate (55 days). The bentonite enhanced the mechanical and physiochemical properties and can be proposed for usage in packaging applications.

Montmorillonite (MMT) is a naturally occurring layered aluminosilicate mineral with a 2:1 clay structure (two tetrahedral sheets sandwiching one octahedral sheet). Singh et al. [44] developed a corn starch biocomposite with glycerol, MMT nanoclay (1.5% *v/v* and 2.5% *v*/*v*), and 2% *v/v* of lemongrass oil-based nanoemulsions (LNE). The incorporation of MMT enhanced the mechanical properties and improved barrier properties. The TS increased from 17.38 to 26.2 MPa, and the EAB decreased from 16.67 to 13.09% due to the interactions between the hydrogen bonds and hydroxyl groups creating an excellent crossing point for providing a uniform distribution of stress and load transfer distribution. Regarding barrier properties, the moisture content and water absorption decreased with the addition of MMT from 14.92 to 14.09% and from 50.11 to 36.45%. These biocomposite materials have promising potential to be used for food packaging to extend the shelf lives of food products.

Another nanoclay that has been used is halloysite nanotube, which has a large aspect ratio, is easily available, and has high functionality, good compatibility, and high mechanical strength. This nanofiller is a multiwall kaolinite nanotube with 1:1 clay layers with lengths typically ranging from 300 nm to 1500 nm. Ren et al. [45] developed a potato starch biocomposite with different types of plasticizers (glycerol and sorbitol) and a halloysite nanotube (3, 5 and 7 wt%). The addition of a halloysite nanotube enhanced the mechanical properties, where the TS increased (glycerol: 2.28 to 3.36 MPa; sorbitol: 9.7 to 10.78 MPa), and the EAB increased with glycerol (26.1 to 34.5%) and decreased with sorbitol (43.3 to 35%). The halloysite showed good dispersion with glycerol due to higher hydrophilic character, and the moisture content decreased with the addition of halloysite (glycerol: 16.7 to 13.8%; sorbitol: 9.6 to 7.5%). The halloysite nanoclay is an effective and promising clay to enhance mechanical properties and can be used as an alternative for replacing non-biodegradable materials in different fields, such as packaging, agriculture, and biomedical applications.

The incorporation of natural fillers consistently enhances the mechanical and barrier properties of bioplastics. From the multiple studies performed, improvements in the tensile strength and water resistance were observed, primarily due to the enhanced interfacial adhesion and the reinforcing effect of filler particles. In terms of barrier properties, the water absorption decreased, and suitable moisture contents made these bioplastics suitable fir packaging, agriculture, and medical applications. Lastly, the natural fillers increased the crystallinity, making the product thermally stable. Natural fillers are recommended to improve starch bioplastic properties.

**Table 1:** Starch-based bioplastics/biocomposites with different natural fillers.

Starch	Fillers	Properties	Ref.
Corn	Pineapple Leaf Microfiber (3, 6, and 9%)	TS increased (48 to 51 MPa) Biodegradation after 28 days showed 82% weight loss for 9%	[46]
Cassava	Pineapple Leaf Microfiber (10, 20, 30, and 40% *w*/*w*)	TS increased (12.94 to 18.37 MPa) EAB decreased (9.23 to 5.73%) Optimal fiber composition, 30%	[47]
Cassava	Bamboo (10% *w*/*w*, untreated, alkali-treated and permanganate-treated)	Optimal TS (3.96 MPa) for alkali Biodegradability after 15 days (5.44% loss untreated, 5.97% loss alkali, and 6.15% permanganate)	[48]
Cassava	Coconut Fiber (5–30%)	Increased TS (3.24 to 11.2 MPa) Decreased EAB (112 to 20%) Decreased water absorption	[49]
Corn	Sugarcane Bagasse (12%)	Increased TS (5.6 MPa) and EAB (37%) Biodegradability weight loss of 30–66% after 35 days	[50]
Corn	Coffee and Risk Husk (1, 5, and 10% *w*/*w*)	Increased TS (7 to 8.7 MPa coffee husk) (7 to 8.1 MPa rice husk) Decreased EAB (12 to 3% coffee husk) (12 to 3% rice husk) Decreased moisture content and high thermal stability	[51]
Sago	Bentonite (1, 2, and 3% *w*/*w*)	TS increased (0.2936 to 0.449 Pa) EAB decreased (149.72 to 73.93%) Biodegradability rate increased with higher bentonite content	[52]
Cassava	Silica Bamboo Leaves (0–5% *w*/*w*)	TS increased (0.53 to 0.75 MPa) EAB increased (0.16 to 0.28%)	[53]
Arrowroot	Arrowroot Fiber (2, 4, 6, 8, and 10% *w*/*w*)	Increased thermal stability (315 to 323 °C) Biodegradability after 12 days, 10 wt% composted 100%	[54]
Cassava	Oil Palm Mesocarp Fiber (3.5, 7, and 14% *w*/*w*)	Increased TS (0.3 to 0.8 MPa) Decreased EAB (79 to 45%) Thermal stability	[55]
Corn	Nile Rose Residues (0, 20, 40, 60, and 80 wt%)	Increased TS (11.7 MPa 40 wt% and 18 MPa 60 wt%) Decreased EAB	[56]
Sugar Palm	Sugar Palm Fiber (0.1, 0.2, 0.3, 0.4, 0.5, and 1.0 wt%)	Increased TS (4.8 to 10.68 MPa) Decreased EAB (38.1 to 25.38%)	[57]
Corn	Walnut Shell Flour (0, 30, 40, and 50 wt%)	Increased TS (9.75 to 16.75 MPa) Decreased water absorption and increased biodegradability (25% weight loss)	[58]
Corn	Corn Husks (5, 10, 15, and 20% *w*/*w*)	Increased TS at 10 wt%. (3.8 to 10.12 MPa), decreased 20 wt% (5.15 MPa) Increased EAB (72.35 to 239.76%)	[59]
Potato	Pine Rosin (0–30%)	Increased TS at 15% (6.28 to 10.19 MPa), decreased to 5.9 MPa at 30% Lowest water absorption 15% (53.5%) and thermal stability	[60]
Corn	Wood (0, 0.27, 0.54, and 0.81 g/g)	Increased TS (6.52 to 12.51 MPa) and EAB (2.60 to 9.43%)	[61]
Corn	Okra Stalk (0–25%)	Increased TS (11.26 to 19.04) and EAB (0.13–5.14%)	[62]

TS: tensile strength; EAB: elongation at break.

### 2.2. Essential Oils (EOs)

Essential oils are a group of volatile aromatic compounds belonging to plant families. They can be obtained from plant parts like leaves, bark, stems, seeds, and flowers. Essential oil is added to the material to enhance antimicrobial properties. The usage of EOs can reduce the cost while giving the product higher mechanical properties. The antimicrobial behavior of EOs is ascribed to the presence of mono/sesquiterpenes, hydrocarbons, and phenolics. These bioactive compounds interact with the bacterial cell wall’s polysaccharides, fatty acids, and/or phospholipids, which results in the loss of irons and cellular contents [63]. The use of essential oils has gained momentum in the field of food packaging due to its antimicrobial properties, and they can extend the shelf lives of products [64]. The findings are summarized in Table 2.

Souza et al. [65] developed corn starch bioplastic films with nanocellulose-stabilized pickering emulsions of three essential oils, namely cinnamon, cardamon, and ho wood, at 2 and 5 wt% concentrations. Ho wood has the highest TS (12.9 MPa) and EAB (26.9%), and cardamon has the lowest mechanical properties (TS 2.4 MPa and EAB 9.1%). The ho wood essential oil showed strong chemical interactions and thermal stability, giving it potential to be used for biological activities and be applied in packaging materials, with further studies needed to evaluate biodegradability.

A recent bioplastic was developed using palm trunk starch. Hernando et al. [66] mixed 10 g of palm starch and 25% (*v*/*v*) of citric acid oil palm solution with varying amounts of glycerol (10%, 20%, and 30% *v*/*v*). As the amount of glycerol increased, the tensile strength and young modulus decreased, and the elongation at break increased. The tensile strength dropped from 7.71 to 5.73 MPa, and the lowest tensile strength was seen at 30% *v/v* 4.03 MPa. The stiffness of the film reduced from 42.54 MPa for 10% *v/v* to 37.21 and 34.98 MPa with 20% *v/v* and 30 *v/v* glycerol, respectively. in regard to elongation, the elongation increased significantly from 18.07% to 26.47% with an increase in the glycerol content by 10–20%. When glycerol increased from 20% to 30%, the elongation increased from 26.47% to 49.37%. After testing the mechanical properties, the samples underwent biodegradability testing. After 9 days, the bioplastic had the highest weight reductions of 34.54, 32.54, and 30.77% for 10–30% *v/v* glycerol, and after 15 days, it had maximum weight losses of 92, 92.85, and 94.73%. The bioplastic with 30% *v/v* of glycerol demonstrated the ability to have superior qualities, being more environmentally friendly and cost effective; thus, this bioplastic can be used as a replacement for industrial packaging.

Enidiok et al. [67] used chia oil, ginger starch, and feather keratin to develop a biocomposite. The biocomposites contained five films with the same amounts of starch, glycerol, and keratin and different amounts of chia oil (0, 0.5, 1.0, and 1.5 mL). The addition of 1 mL of chia oil increased the film thickness to 0.22 mm and reduced the moisture content (12.76%), water solubility (56.98%), and transparency (65.4%). The tensile strength of the biocomposites increased by 1.75%, 20.2%, and 23.6%, respectively, when compared to the starch keratin biocomposite without chia oil, and the elongation at break decreased by 35.4%, 45.4%, and 47.4% when compared with the starch keratin biocomposite without chia oil. Apart from performing the characterization of the biocomposite, we took this study a step further by coating the biocomposites over fresh tomato. The tomatoes were submerged into each composite film and then air-dried for a duration of 6 h at 25 °C, and lastly the tomatoes were placed under laboratory conditions at room temperature with a relative humidity of 69% and a light/dark cycle of 12 h for 21 days with an assessment of the post-harvest quality at regular intervals at 3, 6, 9, 12, 15, 18, and 21 days. From the generated data, it is seen that the addition of chia oil to the ginger starch–feather keratin biocomposite showed significant effects on the physiochemical, mechanical, thermal, and preservative properties. The chia oil biocomposites improved the weight loss of the coated tomatoes and had low effects on the pH, total soluble solids, and ripening index; it also lowered oxidase activity, delaying the ripening of tomatoes and increasing their shelf life.

Criollo-Feijoo et al. [68] developed a bioplastic with 2% bagasse cassava starch–water solution with different amounts of oregano essential oils (1, 2, and 3% of total solution) and 0.5% glycerol. We developed a bioplastic with a 100% total ratio of essential oil, starch, and glycerol. For example, 2% of cassava starch and 0.5% of glycerol were mixed with 97.5% of water instead of the original volume. The films with 3% oregano essential oil showed total inhibition against *S. aureus* (57.81 mm) and *E. coli* (21.98 mm) and had higher thickness (164.7 µm), humidity (11.81%), solubility of the films (30.27%), water vapor permeability, and strain at break (37.9%) but decreased tensile strength (0.39 MPa), which shows that these films can be used as an active packaging material.

Carvacrol is an essential component of oregano essential oil which exhibits antimicrobial activities. Mao et al. [69] developed a potato starch film with different concentrations of carvacrol (10, 20, and 30% *w*/*w*). The incorporation of carvacrol weakened the mechanical properties of the films from 29.5 to 7.5 MPa for the TS and from 20 to 6% for the EAB and showed antimicrobial properties against *E. coli* (28.6 mm) and *S. aureus* (57.2 mm), where the inhibition zone increased with an increasing concentration of carvacrol. Despite its weak mechanical properties, carvacrol was shown to have potential to be used as a coating for fruits and vegetables.

Peppermint essential oil (PEO) is growing in the food industry, extending the shelf lives of fruits and vegetables due to its sensory and antimicrobial properties. Srivastava et al. [70] developed a biocomposite with corn starch, glycerol, and sorbitol with varied amounts of rice husk fiber (10, 20, 30, 40, and 50% *w*/*w* of starch). PEO was added to the 10% *w*/*w* rice husk fiber biocomposite at varied amounts (1, 2, 3, 4, 5, and 6% *w*/*w*). The biocomposite with 10% rice husk fiber and 1% PEO had high moisture content (23.85%) and water solubility (36.36%), the highest TS (2.25 MPa), and a low EAB (14.5%). As the PEO increased, the TS decreased, and the EAB increased. All the reinforced biocomposites were biodegradable within 35 days. In regard to the antimicrobial assay, the incorporated PEO demonstrated the presence of an inhibition zone, where the size increased with a higher percentage of PEO. The incorporated 1% PEO showed an inhibition zone against Gram-positive (*S. aureus* 6 mm) and Gram-negative bacteria (*E. coli* 6 mm). Lastly, a shelf life assessment was carried out on bread sealed with the biocomposite in which physical microbial growth was not found due to the release of bioactive components like menthol, creating a microenvironment inside the package. Overall, the developed biocomposite film with risk husk fiber and PEO has the potential to be used as a packaging material and increases the shelf life of food like bread for 30 days, making it a great choice for preserving food without chemical additives.

Many studies involving adding essential oils into bioplastics and biocomposites have been conducted, but very few have been conducted on emulsification. Emulsification helps improve the dispersion of essential oils, improving the properties of biocomposites. Yang et al. [71] used Zanthoxylum bungeanum essential oil (ZBO) to develop corn starch-based biofilms with three kinds of emulsifiers: Tween 80 as a small molecule surfactant; sodium caseinate (CAS), whey protein isolate (WPI), and gelatin (GE) as macromolecule emulsifiers; and whey protein isolate fibril (WPIF) as a particle emulsifier (emulsions ZBO 1.0% and emulsifiers 0.1% (g)). The WPIF had a higher TS (8.6 MPa), and Tween 80 had the highest EAB (66%) compared to the control sample. The moisture contents and total soluble matter were lower with emulsifiers due to the addition of EO increasing the hydrophobicity, and the EO reduced the thermal stability due to a decrease in temperature degradation. In terms of antibacterial properties, the CAS and Tween 80 films showed better antibacterial properties against *S. aureus*, and CAS and ZBO showed antibacterial properties against *L. monocytogenes.* To summarize, this work showed that macromolecule emulsifiers exhibited the best mechanical and moisture barrier properties and the best antibacterial properties against *S. aureus* and *L. monocytogenes*, showing their potential to be used in green food packaging materials.

Parada-Quenaya et al. [72] developed potato starch bioplastic films with Stipa obtusa microfibers (MFSO) (0.15, 0.35 and 0.55% *w*/*w*) and eucalyptus essential oil (EEO) (0, 0.17, and 0.43% *w*/*w*) using tape casting. The addition of EEO showed a significant decrease in elastic modulus from 286.18 to 16.92 MPa.

Castro et al. [73] developed a chitosan/cassava starch biocomposites filled with bentonite clay/GEO particles with different concentrations of ginger essential oil (0.5, 1, and 2 wt% dry mass). The results showed that the addition of ginger essential oil increased the TS (7.5 to 9.5 MPa) and EAB (11 to 17%) for concentrations of 0 to 1% but decreased for a 2% GEO concentration (to 6 MPa and 7%) due to the effective dispersion of filler particles in the polymer matrix. The bentonite clay/GEO filler increased the antimicrobial properties of the film against *S. aureus* (3.8 mm) and *E. coli* (3.65 mm) for a concentration of 1%. The developed films enhanced the mechanical properties and antimicrobial properties, showing its potential for use in edible films.

Yang et al. [74] used silylated starch, cellulose, glycerol, acrylated epoxidized soybean oil (AESO), and Tween 80 to develop a bioplastic and cured it for 2 h under different temperatures (80, 100, 120, and 140 °C) to promote the reaction between silylated starch and AESO. The AESO acts as a crosslinker. The tensile strength and elongation at break of the bioplastic without AESO were 8.8 MPa and 7.22%, and they reduced slightly with the addition of AESO at 80 °C due to the poor dispersion of AESO in the bioplastic. As the curing temperature increased from 80 to 120 °C, the tensile strength increased, revealing that the interaction between silyated starch and AESO was enhanced. Lastly, the tensile strength reduced when the sample temperature increased to 140 °C. As for the elongation at break, it decreased as the curing temperature increased. The sample at a curing temperature of 120 °C presented the most satisfactory structures, making it beneficial for packaging applications even though there is weak effect of curing temperature on the interaction of modified starch and AESO.

In continuation of their previous work, Yang et al. [75] analyzed the effects of silane hydrolysis time on the physiochemical properties of starch-based epoxidized soybean oil (ESO). The 3-aminopropyl trimethoxy silane (APTES) was dispersed in distilled water and hydrolyzed at 50 °C for 0–24 h with NaOH and starch. Once the mixture was neutralized, it was washed with distilled water and then oven dried for 24 h. The silylated starch samples were based on the hydrolysis time of APTES, namely 0, 1, 2, 4, 8, and 24 h. The bioplastics were produced by blending microcrystalline cellulose, glycerol, and silylated starch in distilled water until gelatinized. Tween 80 and ESO were incorporated into the mixture to develop the bioplastic. This work focused on mechanical properties, thermogravimetric analysis, and enzymatic degradation. The most desirable hydrolysis was 2–4 h as it showed the highest elongation at break and a tensile strength of 8.69 MPa. The water resistance properties were not improved by the silylation of starch. The bioplastic materials maintained excellent biodegradability, and APTES hydrolysis for 4 h presented more satisfactory structural, thermal, and tensile properties for packaging applications.

Enwere et al. [76] used wild cocoyam starch to develop bioplastic films containing gelatine, glycerin, vegetable oil, and vinegar in which the composition of the materials varied with the seven different samples produced. The vegetable oil was used to test whether it is suitable to be used as a plasticizer. The samples were tested to analyze the mechanical properties, moisture content, water solubility, water absorption, biodegradability, and structural analysis of the films. From the results obtained, the sample with the highest tensile strength and elongation at break of 6.5 MPa and 77% and low moisture content (2.4%), water absorption (20%), and water solubility (49%) was the bioplastic film with 2 g of gelatine, 1 mL of vinegar, and 3 mL of glycerin. The cocoyam bioplastic aligns with commercial packaging standards due to the exceeding film thickness of 270 µm and strength and flexibility but with a limitation of thermal stability.

Recent studies have shown that essential oils are beneficial natural additives for biocomposites. They enhance antimicrobial and mechanical properties, helping to extend the shelf lives of biodegradable materials. Additionally, essential oils improve flexibility, making biocomposites more suitable for packaging and medical applications.

**Table 2:** Essential oils in starch-based bioplastics.

Starch	EO (%)	Antimicrobial Activity	Properties	Ref.
Amphiphilic	Cinnamon (0.25, 0.5, and 1%)	*E. coli* (43.8–61%) *S. aureus* (65.9–80.9%)	Increased TS (11.21 to 22.09 MPa) and EAB (16.4 to 26.79%), 70% biodegradable after 28 days	[77]
Corn/Wheat	Lemon (0.5, 1%, and 2%)	*E. coli* (45.46%) *S. aureus* (47.72%)	TS decreased by 28.41%, EAB increased by 19.82%	[78]
Millet	Clove (0–3% *w*/*w*)	*E. coli* (23 mm), *S. aureus* (18 mm), *P. aeruginosa* (24 mm), *Enterobacter* sp. (27 mm), *B. cereus* (20 mm) and *Trichoderma* (14 mm)	Increased thickness and EAB (9.3 to 5.67%), decreased TS (10.52 to 6.25 MPa)	[79]
Corn	Orange (0, 0.3, 0.5, and 0.7 µL/g)	*S. aureus* (68%) *L. monocytogenes* (80%) growth reduction	Increased MC and water solubility, decreased TS (5.11 to 2.4 MPa) and EAB (64.58 to 15.25%)	[80]
Potato	Lavender (2, 4, and 6%)	*S. aureus* (24.5 mm) *E. coli* (15.1 mm)	Increased thickness; decreased water solubility, absorption, and TS (70.2 to 24.89 MPa)	[81]
Chitosan	Rosemary (0.5%)	*B. subtilis, E. Coli, and L monocytogenes*	Improved water barrier properties, TS (25.95 MPa), and EAB (14.87%)	[82]
Cassava	Tea Tree (0.08, 0.8, and 1.5% *v*/*v*)	*S. aureus* (68%) *C. albicans* (64%)	TS increased (3.73 to 8.34 MPa) and decreased by 1.5% (3.03 MPa) EAB decreased	[83]
Tapioca	Caraway (0.5, 1, 2, and 3% *w*/*w*)	*B. cereus* (29.83 mm), *E. coli* (10.33 mm), *P. aeruginosa* (12.63 mm)*, S. aureus* (26.3 mm)	TS decreased (15.23 to 12.62 MPa), EAB decreased (27.84 to 20.94%)	[84]

TS: tensile strength; EAB: elongation at break; MC: moisture content.

### 2.3. Nanoparticles

Nanoparticles, which are particles ranging in size from 1 to 100 nm in diameter, have gained significant attention in recent years for their innovative incorporation into biocomposites, enhancing their mechanical, thermal, and functional properties. The most used nanoparticles are zinc oxide (ZnO), silicon dioxide (SiO_2_), titanium dioxide (TiO_2_), and calcium carbonate (CaCO_3_), which can block UV radiation and act as antibacterial agents [85]. Additionally, carbon-based nanoparticles such as graphene derivatives and carbon nanotube have been widely studied for their reinforcement effect on the polymer matrix. The findings are summarized in Table 3.

Iacovone et al. [86] developed a biocomposite with TiO_2_ nanoparticles (NP) using cassava starch, glycerol, and distilled water using the extrusion method at 80 rpm and 120 rpm with and without TiO_2_NP. The samples developed at 80 rpm showed higher tensile strength (3.2 MPa) but a lower elongation at break (57%). The incorporation of TiO_2_NP decreased the elongation at break by 16% for 80 rpm and increased it by 13% for 120 rpm. Also, the moisture content slightly decreased water resistance for both 80 rpm (18%) and 120 rpm (19%). Due to its improved properties, the TiO_2_NP biocomposite at 120 rpm underwent biodegradability testing. The incorporation of TiO_2_NP accelerated the biodegradation rate and increased the final degradation percentage (106%). TiO_2_NP in cassava starch biocomposites showed potential as a packaging material for its fast-degrading properties under industrial composting.

Santos et al. [87] developed a potato starch bioplastic incorporating CaCO_3_. The CaCO_3_ was separated from organic eggshell membranes using the air floatation method and consolidation process of mixing starch (10, 20, and 30% *w*/*w* eggshell dry weight) with the eggshell suspension, and then it was frozen and dried and then ground to a powder. The eggshell and eggshell/starch mixture were melt-mixed with low-density polyethylene (LDPE). The film with 30% starch had the lowest density and was further tested. When 30% of starch and 50% eggshell were added, the TS and EAB decreased from 20.5 MPa to 14.5 MPa and from 14.1% to 5.8% when compared to the LDPE-COM control film, with increasing rigidity. The starch consolidation of CaCO_3_ revealed to have potential in developing lightweight fillers for LDPE plastics.

Oluwasina et al. [88] developed a bioplastic with physico-electrical properties. They used cassava starch and waste from dry cell batteries. The bioplastic films were produced with varying starch compositions, acid-treated Luffa cylindrica cellulose (ALC-cellulose), and graphene oxide (GO). The bioplastic films had a control bioplastic with just starch and varying amounts of ALC-cellulose and GO. This paper tested the mechanical properties and electrical properties. As the concentrations of GO and ALC-cellulose increased, the moisture content (MC) decreased from 8.4% to 3.5%. For the mechanical properties, ALC-cellulose contributed more to the increase in tensile strength than GO due to the creation of intermolecular hydrogen bonding between the starch base and ALC-cellulose. The tensile strength increased from 0.98 to 1.42 MPa. The ALC-cellulose increased the elongation at break as compared to the bioplastic with only GO. The highest elongation was 78.40%. The electrical conductivity of the bioplastic with GO and ALC-cellulose showed the highest conductivity of $14.7\times 10^{-3}\;S/m$ as compared to the other films with starch only and GO. This paper showed that discarded battery rod graphite and waste Luffa clyindrica L can be used as raw materials.

Nanoparticles not only enhance mechanical and physiochemical properties, but they also present antimicrobial activity. Arezoo et al. [89] developed sago starch films with different concentrations of TiO_2_ nanoparticles (0, 1, 3, and 5% *w*/*w* sago starch) and cinnamon essential oil (0, 1, 2, and 3% *w*/*w* sago starch) and a mixture of glycerol and sorbitol (1:3) 40% *w*/*w*. The increased amounts of TiO_2_ and CEO led to significant decreases in water solubility, moisture content, and water absorption. Likewise, the TS decreased and the EAB increased. The starch film with 5% TiO_2_ had a higher tensile strength and lower elongation at break, and as the concentration of CEO increased, the TS decreased and the elongation at break increased. Lastly, the 5% TiO_2_-2% CEO showed the highest inhibition zone for antimicrobial properties against *S. thyphimurium* (5 mm), *E. coli* (6 mm), and *S. aureus* (7.5). The higher the concentrations of TiO_2_ and CEO, the higher the inhibition zones. TiO_2_ improved the mechanical and barrier properties while showing antimicrobial properties, and combining it with CEO showed its great potential for use in active edible film in food industries.

**Table 3:** Nanoparticles in starch-based bioplastics.

Nanoparticles	Starch	Properties	Ref.
ZnO	Cassava (1, 2, 3, 4, and 5% *w*/*w*)	MC decreased, TS increased to 10.29 MPa, and EAB decreased to 5.69%; biodegradation occurred at 7 days	[90]
	Banana (1, 3, and 5% *w*/*w*)	TS increased from 2.5 to 36 MPa, and EAB decreased from 28 to 8%; degradation time was 90 min	[91]
SiO_2_	Potato (0–1.5%)	Increased EAB (from 52 to 70%) and decreased tensile strength (from 1.1 to 0.2 MPa); biodegradation occurred at 5 days	[92]
	Corn (0–1.5%)	Increased EAB (from 59.2 to 78.9%) and decreased TS (from 1.05 to 0.6 MPa); biodegradation occurred at 40 days	
TiO_2_	Corn	Increased TS (from 3.55 to 3.95 MPa) and decreased EAB (from 88.1 to 62.5%); biodegradation with 64% weight loss occurred at 30 days	[93]
CaCO_3_	Potato (10, 20, 30, 40, and 50% *w*/*w*)	Increased TS (from 30 to 45 MPa) and decreased EAB (from 20 to 15%) Decreased water absorption	[94]
	Cassava (2, 4, 6, 8, and 10% *w*/*w*)	Increased TS by up to 4% (3.25 MPa) and decreased EAB (from 53.14 to 26.5%) Decreased MC, thermally stable	[21]
GO	Potato	Optimal ratio of starch:chitosan (75:25) Enhanced mechanical properties (TS 26 MPa), water resistance (<10% weight loss), and electrical conductivity (3.8 × 10^−3^ S/m)	[95]
Copper	Corn (0.25, 0.5, 0.75, 1, and 5% *w*/*w*)	Increased TS (from 1.6 to 1.75 MPa) Decreased EAB (from 26 to 8%) Antimicrobial against *S. aureus* and *E. coli*	[96]

TS: tensile strength; EAB: elongation at break; MC: moisture content.

### 2.4. Polymer Blends

Polylactic acid (PLA) is a linear aliphatic thermoplastic polyester derived from lactic acid from the fermentation of renewable and biodegradable sources like corn starch and raw materials with a high sugar content. PLA is biodegradable, renewable, and biocompatible, but it is brittle and has a low oxygen barrier. Both PLA and starch have opposite mechanical and barrier properties, making them insufficient for compatibility. To solve this issue and prevent phase separation, compatibilizers are added to promote polymer interfacial interaction, improving the properties of the blend. The findings are summarized in Table 4. Collazo-Bigliardi et al. [97] used grafted poly(ε-caprolactone) (PCL) as a compatibilizer to develop a corn starch/PLA biocomposite with twelve samples made with pure starch, pure PLA, and starch/PLA (20 and 40%) blends with PCL (2.5 and 5%). The film containing 20% PLA and 5% PCL had good TS and EAB. The incorporation of PCL decreased oxygen permeability by 40%, showing great potential for food packaging for products like dry or partially dehydrated products.

Polylactic acid (PLA) has the advantages of being biodegradable and transparent, with good mechanical properties, and it is safe for food packaging but is limited due to its high cost. To reduce the cost, PLA is blended with low-cost biopolymers or biofillers that modify the properties of the resulting composites. Estrada-Giron et al. [98] took advantage of developing a PLA/*Dioscorea remotiflora* starch biocomposite, where 50 g of PLA was mixed with different components of starch (7.5, 15, 22.5, and 30 wt% (dry basis)) using compression molding, which is a process that avoids applying high shear stresses to the starch granules. According to the results obtained, the addition of starch affected both the flexural and tensile strength. As the amount of starch increases, the flexural and tensile strength decrease due to the reduction in adhesion between the blended materials. The biocomposite with 7.5 wt% had the highest flexural and tensile strengths of 68.5 MPa and 76.54 MPa. The uniqueness of this work lies in its analyses of the biocomposite, including water absorption kinetics and dynamic mechanical analysis. PLAs blended with high-amylose starch exhibited larger water uptake, faster swelling kinetics, a higher crystallinity, and improved flexural modulus as comparted to PLA alone.

Another study was conducted, taking advantage of PLA and mixing it with starch. Baniasadi et al. [99] developed a PLA/potato starch biocomposite by mixing PLA granules with varying concentrations of n-octadecyl isocyanate (ODI) potato starch substance (10, 20, 30, 40, and 50 wt%) using the injection molding method to produce the specimens. A solvent-free method was used to graft the ODI on the surface of starch to enable the compatibility of the PLA matrix. The increase in the proportion of ODI-starch enhanced the tensile strength, impact strength, tensile strain, and toughness, with the 20 wt% biocomposite having high tensile strength (52.3 MPa) and strain (19%). Furthermore, the biocomposite with 50 wt% was selected for filament production in 3D printing due to its high starch content even though it had the lowest tensile strength (35.3 MPa) and strain (3%). A variety of shapes were successfully printed, like simple cups, dog bone specimens, 3D curvatures, and intricate grid geometries in which there were no issues with the printing process, indicating excellent stability of the extruded filaments and strong adhesion between layers with smooth surfaces. The inspiration of using 3D printing shows a significant promise for the future of sustainable materials, showing the potential for a reduced carbon footprint as compared to petroleum-based plastics.

Moghaddam et al. [100] developed a biocomposite utilizing PLA and polybutylene succinate (PBS) using design of experiments with three independent variables, PLA:PBS (50:50), corn starch, and what straw. The concentrations had a five-level variance in the ranges of 30–70 for PLA:PBS, 30–60 for cornstarch, and 0–8 wt% corn starch for wheat straw regarding the mechanical properties, and the equilibrium moisture content was determined using response surface methodology. The biocomposite film was prepared by mixing dried corn starch and wheat straw with the PLA:PBS blend along with Joncryl and zinc stearate. The response surface methodology showed significance with the R^2^ of the elastic modulus (EM), elongation at break (EAB), impact strength (IS), and equilibrium moisture content (EMC) being equal to 0.95, 0.97, 0.97, and 0.99, which shows that the model is accurate enough to predict the responses. The optimal values predicted by the models for PLA:PBS, corn starch, and wheat straw were 48.2 wt%, 45.4 wt%, and 6.4 wt%, respectively; the EM, EAB, IS, and EMC were 80.8 MPa, 11.4%, 2 kJ/m^2^, and 4.1%. The experimental results for the EM, EAB, IS, and EMC were 76.9 MPa, 10.9%, 2.1 kJ/m^2^, and 4.4%, showing no significant difference between the experimental data and predicted values. As seen from the optimal results, a disposable container was prepared using injection molding with a biodegradability rate of 71.1% in the fifth month, showing that it is inexpensive to develop due to having more natural polymers in the biocomposite.

Kurup et al. [101] studied the impact of processing parameters on the compatibility and performance of PLA/tapioca starch biocomposites. PLA and tapioca starch were combined with maleic-grafted PLA as coupling agent and epoxidized palm oil as a plasticizer in ratio of 65.7:27.9:4.9:2, and response surface methodology was used to find the optimization of impact of processing parameters. The Box–Behnken design was used with three-level factors, three independent variables (injection temperature, injection pressure, and injection speed), one measured response (tensile strength), and 17 experimental runs. The injection temperature had the most significant influence on tensile strength in which the highest tensile strength was at around 180 °C, and a higher injection speed decreased the tensile strength caused by shear-induced degradation. The model estimated a tensile strength of 25.808 MPa using an injection temperature of 181 °C, an injection pressure of 40 MPa, and an injection speed of 300 mm/s, and the experimental data show a tensile strength of 25.344 MPa, which falls within the predicted value with error of just 1.78%. The optimized biocomposite had a maximum water absorption rate of 1.95% after 10 days, an elongation at break of 16%, which is lower than PP but higher than PLA, and a biodegradation rate of 2.84%, showing great potential as short-term packaging material. This paper focused more on optimization processing parameters for the industrial scale rather than different ratios of the biocomposites, showing potential for industry use.

Oluwasina et al. [102] developed a bioplastic of starch and bis(2-hydroxyethyl) terephthalate (BHET). The polyethylene terephthalate (PET) water bottles were crushed into smaller particles and washed with NaOH solution of a 1:10 solid-to-liquid ratio, followed by rinsing with hot water and drying. The bioplastic was developed by adding BHET (1 g, 425 µm) and distilled water (50 mL) while being heated at 90 °C with the addition of glycerin. The mixture was stirred continuously until 100 °C was reached and then casted into a Teflon mold, and the film was then oven-dried (70 °C). Different films were developed with the addition of 0%, 20%, 40%, 60%, and 80% of BHET (5 g of starch). These films were compared to the bioplastic with starch only. The results showed a low moisture content and water solubility decreasing from 40.54% to 23.45%, showing that bioplastics produced with BHET can withstand water-related challenges. The mechanical properties were significantly affected by the addition of BHET, which affected the tensile strength, elongation, and Young’s Modulus. The 40% BHET concentration resulted in the best mechanical properties because it had the highest tensile strength (2.59 MPa), stable elongation, and Young’s modulus.

Mohammed et al. [103] developed a wheat starch/polyvinyl alcohol (PVA) biocomposite with different concentrations of sugar palm fibers (3, 6, 9, and 12 wt% of starch). The blending of starch and PVA improved the mechanical properties. The addition of PVA reduced the moisture content (9.17%) but increased it with the addition of fibers (10.24%). The improvement in tensile strength occurred at 9 %wt. fiber, which was 12 MPa, but the 3 %wt. concentration had the highest EAB at 66.3%. Reinforcing starch/PVA has shown improvement; the 9 %wt concentration showed the best properties as it showed more resistance to water uptake, thermal stability, and good mechanical properties, with no recommendations for use in industrial applications.

**Table 4:** Compatibilizers for starch/PLA biocomposites.

Starch	Compatibilizer	Properties	Ref.
Cassava	Oligo (lactic acid) (1, 2, 3, and 5 wt%)	Decreased TS (from 45 to 21 MPa) Increased EAB (from 5 to 35%) Improved extensibility, water and oxygen vaper barrier properties, and thermal stability	[104]
Yam	Epoxidized sesame oil (1.5 and 3 wt%)	Increased deformation and decreased TS Thermal stability	[105]
Cassava	Glycidyl methacrylate (1 wt%)	Decreased TS (from 37.4 to 23.3 MPa) Increased EAB (from 3.6 to 8.6%)	[106]

TS: tensile strength; EAB: elongation at break.

## 3. Current Industrial Applications

Renewable biomaterial resources derived from bioplastics and biocomposites have emerged as an enticing research area for academia and industry, offering a promising sustainable solution to the global plastic waste crisis. Driven by growing social and environmental awareness, the industrial sector has undergone significant changes in recent years, with manufacturers increasingly adopting cradle-to-grave product designs and techniques. This shift has led to the integration of bioplastics and biocomposites derived from renewable biomaterials [107,108].

**Figure 3:**
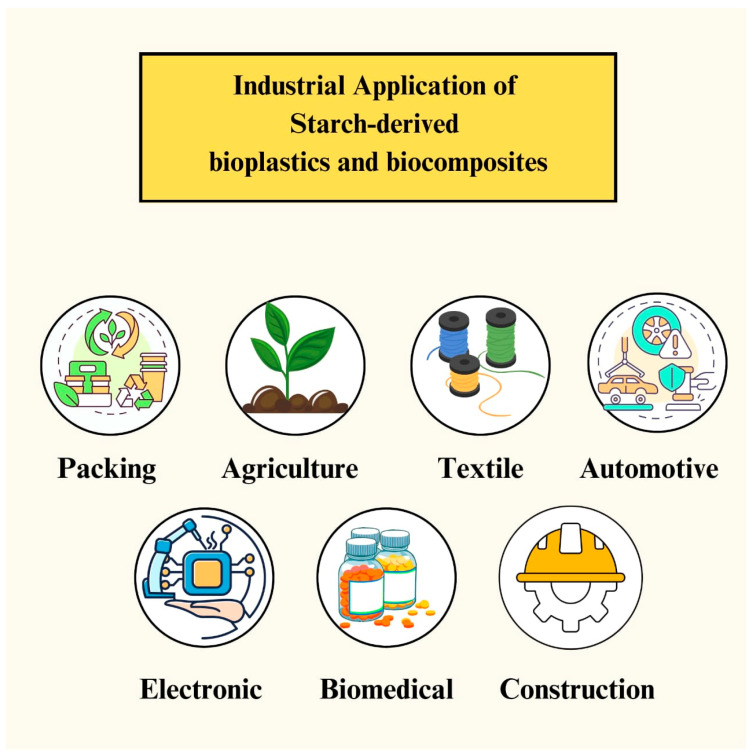
Industrial applications of starch-derived bioplastics/biocomposites.

As discussed in this review, starch-derived bioplastics and biocomposites dominate industrial applications due to their low cost, abundance, and excellent film-forming properties. Additionally, their distinct physical properties, including biodegradability, renewability, and adaptability, make them highly suitable for various industries. These biomaterials are applicable in packing, agriculture, automotive, textile, biomedical, consumer goods, and electronics applications, providing a sustainable, eco-friendly alternative that aligns with environmental and circular economy concerns, as shown in Figure 3 [108].

This section provides an in-depth examination of the industrial implementation of starch-based bioplastics, highlighting trends, recent advancements, challenges, and a future perspective on their adoption.

### 3.1. Packaging Industry

The packing industry is considered the largest consumer of starch-based bioplastics, accounting for over 50% of the global bioplastic market. These materials offer excellent, unique physical properties in their biodegradability as they have moderate mechanical strength and can act as barriers against weather conditions. They are usually applied in biodegradable wraps, bags, and food containers [109].

Recent advancements have been implemented in these biomaterials, where nanoparticles such as nanoclays, cellulose nanofibers, and graphene oxide enhance their mechanical strength and barrier properties. The studies by Tang et al. [110] and Martins et al. [111] demonstrated that nanoclay is significantly incorporated into starch-based films, which improves their tensile mechanical strength and water resistance.

Likewise, other studies showed the use of biopolymer blends, such as starch–polyvinyl alcohol composites, to improve moisture resistance. Also, others demonstrated that starch-based foams are being developed as an alternative to expanded polystyrene for cushioning and protective packing applications [112]. Further advancements in biodegradation kinetics have also enabled researchers to optimize formulations for specific environmental conditions [113].

A study conducted by Dutta et al. [114] focused on developing starch-based intelligent packing films that integrate natural antioxidants to improve their use for food preservation. The research incorporated rosemary and green tea extracts with starch-based bioplastics, improving the oxidative stability of packaging food products. Similarly, Wang et al. [115] studied starch–chitosan composite films and investigated their antimicrobial properties, showing that they are ideal for for protecting various perishable food items.

### 3.2. Agricultural Applications

These biomaterial films and composites have shown promising applications in the agricultural field, mainly in mulch films, seed coating, and formulating controlled-released fertilizers. These manufactured films enhanced soil moisture retention, reduced weed growth, and improved crop production and yield in terms of biomass [116].

Starch-based controlled-release fertilizers (CRFs) are the trends in innovative applications in recent research. This implies that the main macronutrients and micronutrients in starch-derived hydrogels allow for controlled nutrient release, improving the efficiency of nutrient use and mitigating environmental pollution. Also, scientists have examined the potential of starch-based films reinforced with biochar and lignin to further enhance their structural integrity for improved water resistance, as other advances in crosslinking techniques and polymer blending have further enhanced the mechanical stability of these synthesized agro starch-based biofilms [117]. Shelar et al. [118] studied the use of starch-based biodegradable seed coatings with embedded micronutrients. The findings indicated improved seed germination rates and seedling vigor. This approach is considered a new pathway for sustainable agricultural application, leading to more successful implementation and increased production. Also, Quilez et al. [119] studied the effect of starch-polyGly blends in mulch films, reporting enhanced UV resistance, prolonged soil protection, and increased soil amendments.

### 3.3. Textile Industry

Different biodegradable and nonwoven fibers have been explored in starch-derived bioplastic development. These materials are mainly utilized in single-use hygiene products such as diapers, wipes, and sanitary napkins [120].

Recent industrial improvements have been implemented to advance the mechanical properties of starch-based nanofiber fabrication through advanced electrospinning technology [121]. Khoo et al. [112] demonstrated that starch nanofibers exhibit high porosity and surface area, making them suitable for air and water filtration applications. Others suggested incorporating natural antimicrobial agents like essential oils with chitosan polymer in their matrix to enhance the antimicrobial properties of these fibers. Starch-based coating methods on textiles have also been used to provide hydrophobicity and antimicrobial properties against many microbial strains [122].

On the other hand, Zhara et al. [123] explored the application of starch-based nanofibers in medical textiles, showing that these nanofibers improved moisture management and breathability. Additionally, Biehl et al. [124] studied the starch–protein compound in formulating hybrid fibers for implementation in sustainable fashion applications, revealing a controlled improvement in biodegradability and increasing mechanical and tensile strengths.

### 3.4. Automotive Industry

The automotive industry has embraced starch-based bioplastics to improve fuel efficiency and reduce vehicle weight. Different interior components of vehicles have been created with starch-based biocomposites, like the dashboards, door panels, and seat cushions, using biocomposites combined with natural fibers like flax, hemp, and jute [125].

On the other hand, vehicle seats and headrests are currently made with biodegradable foams made from starch, providing a sustainable alternative to conventional-based foams and offering a light weight. Studies indicate that starch-based nanocomposites can be infused with nanoclays or graphene, which significantly improves the thermal stability and mechanical strength of this nanocomposite and advances the automotive industry’s performance [125].

Oh et al. [126] studied starch-based thermosetting resins for automotive applications, revealing improved heat resistance and impact strength. Thapliyal et al. [127] developed biofiber-reinforced starch composites for reducing carbon emissions and light-weight automotive panels during vehicle operation.

### 3.5. Biomedical Applications

Starch-derived bioplastics are extensively valuable for biomedical applications for their non-toxicity and biocompatibility with controlled degradation properties. Primarily, they are utilized in wound dressing, drug delivery systems, and tissue engineering scaffolds [128,129]. Rai et al. [130] demonstrated the excellent absorption properties and high oxygen barrier properties of biopolymer-based hydrogels, which make them effective in wound-healing applications.

Others showed that starch/nanoparticles have excelled in delivering targeted drugs because their controlled degradation allows for precise medication release. Advances in 3D printing technology have also enabled the fabrication of starch-based scaffolds for bone and cartilage regeneration [131].

### 3.6. Electronic Industry

The electronic industry has adopted starch-based biocomposites for manufacturing biodegradable casing and components. These materials provide an eco-friendly alternative to traditional petroleum-based polymers used as primary components on electronic devices. Starch-based composites reinforced with natural fibers have been used for casing mobile phones, laptops, and electronic accessories [132].

Besides that, other advanced formulations integrate nanoclays and biopolymers with starch-based biocomposites to improve their thermal and mechanical stability. However, large production and material optimization are still challenges that require further research to offer cost-effective solutions [133].

### 3.7. Consumer Goods

Starch-derived bioplastics are increasingly used in disposable consumer goods, including plates, cutlery, and food containers. Different companies, such as Biome Technologies and Novamont, have developed starch-derived bioplastic products that fully degrade in a composting environment, thus reducing the waste accumulation crisis [133,134].

Research conducted by Muñoz-Gimena et al. [135] demonstrated starch-based biodegradability for cosmetics packing, showing improved stability and an extended shelf life. Also, Lepak-Kuc et al. [136] showed that starch derived from potato can be used in synthesizing flexible films for electronic packing, revealing superior mechanical flexibility and biodegradability.

Innovations in processing techniques like injection molding, twin-screw compounding, and reactive extrusion have enhanced these starch-based biomaterials’ mechanical properties and esthetic quality. Even so, researchers are still improving these materials’ stability and usability to overcome the challenges of moisture resistance [137].

### 3.8. Construction

Plastic in construction is commonly used in pipes, insulation, floor coverings, cables, and more. Bioplastic materials have great application potential but come with the challenges of high cost and resistance to different heavy workload conditions [138]. Also, construction material is preferable for conventional plastics as the properties of bioplastics, like mechanical strength and life span, are not guaranteed. With these challenges, researchers developed bioplastic construction materials with the use of reinforcement to improve mechanical properties.

Lignocellulosic fiber-reinforced biocomposites are commonly used in construction products like window frames and doors because of their strong mechanical properties, hydrophobic properties, and biodegradability. Vitola et al. [139] developed a biocomposite from potato starch-based binders and hemp shives, and the mechanical properties improved with addition of both sodium metasilicate and glycerol, resulting in enhanced compressive strength. The results highlight the potential of these modified binders to be used for sustainable building materials.

## 4. Environmental and Economic Impacts

Implementing bioplastic-based materials as a viable, sustainable alternative to conventional petroleum-based plastic materials has recently captured significant concerns in various applications. These escalating concerns are aligned with social and environmental attempts to mitigate the effects of plastic pollution, the climate change crisis, resource depletion, and biodiversity loss. Bioplastic materials, mainly those derived from natural raw resources, are now replacing existing conventional plastic materials in fostering driven economic growth and consumer demands alongside the circular economy approaches. This section provides an in-depth, comprehensive, compelling analysis of the sustainability of bioplastics through life cycle assessments, discussing the biodegradation and recycling process, the circular economy of bioplastics, and the economic feasibility of large-scale products and regulations.

### 4.1. Sustainability: Life Cycle Assessments of Bioplastics

Life cycle assessment (LCA) for bioplastic materials is classified as a primary sustainability tool to assess the environmental footprint of bioplastics, especially their waste and their environmental and social impacts. As is known, these materials encompass different renewable sources to end-of-life disposal using different innovative biodegradation and recycling approaches [140]. Mainly, what is derived from natural raw resources like starch-based polymers and other polymers such as polylactic acid (PLA) are considered unequivocal plastics, which exhibit a lower carbon footprint than conventional plastic materials. A recent study conducted by Mastrolia et al. [141] demonstrated that PLA’s global warming potential is markedly lower than that of other chemical polymer structures like polyethylene and polypropylene materials because it relies on plant-based feedstocks that can actively absorb atmospheric CO_2_, thus lowering the effect of gas emissions on the total global warming potential.

However, assessing the sustainability considerations for these bioplastic materials extends beyond their effect in the mitigation of greenhouse gas emissions. Other factors play an important role in evaluating their sustainability in different fields. For example, for agriculture fields, what type of agricultural practices are conducted in land fields, how does water resource consumption influence the bioplastics used for these practices, and what is its overall environmental performance and sustainability in its implementation and disposal? Lizundia et al.’s [142] study highlights that producing large-scale bioplastic materials at a commercial agricultural scale could intensify competition for arable land and freshwater resources, affecting and threatening food security. Furthermore, the energy-intensive nature of producing certain bioplastic materials can contribute to the overall environmental benefits, mainly if it is counted in the account of the fossil fuel aspect [143]. Consequently, optimizing the selection of agricultural feedstock and integrating renewable energy into production are essential in enhancing the sustainability of bioplastics [144].

### 4.2. Degradation and Recycling

Biodegradability and recyclability for bioplastics have gained significant interest in industries seeking sustainable green material alternatives to replace conventional petroleum-based plastic, which is often discarded into the environment. Various strategies and policies have been explored to manage bioplastic waste, focusing on biodegradation and thermal and mechanical degradation. One study focused on the bioplastics derived from polylactic acid (PLA) and the biodegradation of polyhydroxyalkanoates (PHB), where their actual degradation potential rate is highly dependent on specific environmental conditions, like soil burial, and biodegradation in soil and compositing is considered as an effective disposal method [144,145]. However, there are specific challenges in optimizing the degradation rate to match waste management policies. Some industrial composting facilities provide the necessary optimal heat. Microbial activity exists to degrade and break down PLA effectively into environmentally safe compounds and does not cause any real environmental issues or crises [146]. Even in ambient and less controlled environments like marine settings or landfills, the process of degradation can be dramatically slower than in other places and sites. This discrepancy gap raises serious concerns about the potential longevity for bioplastics to persist in environmental degradation and decomposition under suboptimal degradation conditions [146,147].

Another recent review discusses various bioplastic recycling routes, emphasizing the mechanical recycling approach for mainly PLA-based bioplastic materials through challenges in maintaining material integrity after multiple recycling cycles, limiting their applicability for high-performance products [147]. Thermal degradation techniques, such as pyrolysis, have also been implemented to recover energy and valuable chemical compounds from bioplastic waste [109,147]. It was noticed that this method’s efficiency relies on the type of bioplastic waste from which the polymer structure was used in the synthesis. Besides that, innovative chemical recycling methods, such as hydrolysis and glycolysis, can be used to deconstruct the biopolymer through the depolymerization technique into monomers, facilitating the creation of re-polymerized PLA into virgin-quality PLA [145]. Therefore, prioritizing the development of closed-loop recycling systems for bioplastics is not merely crucial. However, enhancing circular economy initiative demands and strategies is essential. Besides that, it also contributes to significantly minimizing and reducing the environmental impact for a more sustainable future with a truly eco-friendly solution to fight the environmental crisis [146]. Despite these efforts in recycling bioplastic waste, there is still a need to set up more standardized waste management policies and infrastructure and facilities to be able to provide an optimized recycling process for a large scale of bioplastic waste and to cover all valuable materials [144].

### 4.3. Circular Economy and Bioplastics

Integrating bioplastics into the circular economy framework is essential to maximize their environmental and economic benefits and potential. A circular economy seeks to minimize and reduce waste and lessen resource consumption by promoting the principles of reuse, recycling, and regeneration of materials. A life cycle assessment study emphasizes the importance of bioplastic materials in manufacturing products to significantly reduce the dependence on fossil fuels. Thus, the environmental gains can only be realized with the proper disposal of this bioplastic waste through specific biodegradation and recycling approaches [142].

Unlike the traditional linear economic approach of the “take–make–dispose of” model, a circular economy strategy for bioplastics emphasizes sustainable sourcing, an efficient production scale, and responsibility for end-of-life management [120]. Therefore, in order to achieve circularity in bioplastics, it is crucial to develop advanced state-of-the-art recycling infrastructure and industrial compositing facilities that can effectively, efficiently, and perfectly handle biodegradable plastics. Moreover, innovative designs of bioplastics with improved mechanical properties, stability, and chemical recycling compatibility can extend their lifecycle. Also, this will reduce the dependency on virgin materials; therefore, groundbreaking advancements in bioplastic formulations, such as incorporating bio-based additives and reinforcing fibers, can bolster durability and recyclability, further promoting a circular economy [142,148].

### 4.4. Economic Impacts

Different parameters like product costs, consumer demand, and regulatory policies and frameworks markedly influence the economic feasibility of bioplastics. Currently, bioplastics require and face higher production expenses than conventional plastic due to main factors such as intricate feedstock cultivation, fermentation process, and polymerization complexities. Nevertheless, the landscape is changing as advances in bioprocessing technologies and an increased production scale contribute to cost reductions, making bioplastic production more feasible and viable at a large commercial scale [148,149].

The market demands for bioplastics are steadily growing, propelled by growing consumer preferences, their social and environmental commitment to sustainable products, and stringent policies aimed at combating plastic pollution. The European Union’s directives on single-use plastic and extended producer responsibility (EPR) schemes effectively incentivize adopting and transitioning to biodegradable and compostable alternative plastics [150]. In the United States, state-level bans and support for utilizing eco-friendly packing also ignite enthusiasm for bioplastics and further stimulate interest in bioplastics [131,148].

Investment in bioplastic research and development has become more of a focused, driven field for economic impact. Therefore, it is crucial to overcome existing limitations by finding more solutions for these limitations to overcome them and foster economic viability. A recent study by Market Research Future (2023) predicted that the global bioplastics market will reach USD 30 billion by 2030, unveiling vast opportunities in green packing, agriculture, and biomedical fields. Strategic government subsidies, tax incentives, and collaborative efforts between the academic research field and industry will be significant and pivotal in fostering innovation and reducing production costs to enhance the economic competitiveness of bioplastics. This concerted approach will enhance bioplastics’ economic attractiveness and lead to more sustainable, greener solutions to transition to a more sustainable economy [151].

## 5. Challenges and Future Prospects

Many scientists and researchers have developed bioplastics composed of starch, protein, PLA, cellulose, fibers, nanoparticles, and much more, showing their great potential to replace conventional plastics, but there are challenges in developing them. Firstly, developing bioplastics and biocomposites with excellent mechanical and thermal properties, oxygen permeability, gas barriers, and water vapor transmission rates is challenging. It is important to understand the relationships between the formulation and performance of the material, which includes the structure, processing conditions, shape, and applications. There is ample information about their mechanical and thermal properties, but there are several elements that need to be investigated more. These include the interactions between the different components in the formulations of bioplastics and understanding the effect of formulation on the temperature-dependent properties of the material [152,153].

Another challenge is that bioplastics do not solve the pollution issue even though they are an alternative to conventional plastics. It is very important to educate consumers and governments to build effective infrastructure for collection, recycling, and composting. These infrastructures must have the capacity for large-scale biodegradation and by-products of biodegradation, stressing the importance of returning materials to the environment without negative effects [154]. For example, one of the leading bioplastics in the market is PLA-based bioplastics, but degradation only occurs under industrial composting conditions at high temperatures. Industrial composting is a controlled process and requires high temperatures above 60 °C and may take between 90 and 120 days to fully biodegrade, but misuse leads to waste mismanagement [155].

The number of products produced from cellulose, chitosan, lignin, and starch is growing, but these are limited by the availability of resources and manufacturing processes, which are sometimes not environmentally friendly. The green technology available to modify these sources pose a great challenge in terms of economic viability. For example, it may seem straightforward to develop a starch- or cellulose-based bioplastic, but when it comes to industrial-scale or source extraction, the price estimates for the process, source, and additive are not established adequately, which affects the final price of the bioplastic. Nevertheless, marginal production costs can be reduced by improving chemical technologies and processes limiting price increases. However, these enhancements require higher capital investment than what is currently in place. Due to this financial constraint, many companies opt to continue producing conventional plastics instead, and it also prevents them from being motivated to invest in bioplastics [153,156].

Bioplastics face a challenge from the high cost of being marketed as compared to conventional plastics. They are limited in production and mechanical and barrier properties. Bioplastics have an issue competing with conventional low-cost plastics due to their deficiencies in their formulation, processing, and end-of-life disposal. Currently, there are cheap options available, but they have not completely displaced petroleum-derived plastics due to economic reasons for the company and consumers. Based on the time spent on developing bioplastics by scientists and researchers, the use of bioplastics can reduce the environmental burden, but completely transitioning from petroleum-based plastics to bioplastics can create new burdens with agriculture and competition for food resources [155,157].

The future of bioplastics looks promising, driven by the need for sustainable and economically friendly materials. The key is for consumers to be educated about how to recycle and degrade bioplastics with the help of the government to spend more funds on building effective collection, recycling, and composting infrastructure. Many governments, plastic companies, and consumers are starting to support the circular economy, which accelerates the potential of bioplastics due to their bright future in packaging. The rise in consumer and industrial demand for home-compostable and/or fully marine-biodegradable bioplastics and polymers that are degradable in any environment are expected to leave the future bioplastic market with the exclusion of PLA due to it only being industrially compostable. Recently and in the future, bioplastics will begin to develop from renewable sources such as PHA or other plant-based bioplastics and become commercialized, with innovative ways of improving their mechanical, physiochemical, and thermal properties to make them sustainable. Marine algae are a promising alternative to develop bioplastics due to their abundant biomass and ability to degrade plastics through toxins or enzymes [158]. The future of bioplastics looks promising, driven by technological advancements, economic incentives, and policy reforms aimed at tackling plastic-related challenges. With continued innovation and strategic support, bioplastics can play a pivotal role in reducing the environmental impact and advancing the goal of carbon neutrality [153,155,159].

## 6. Conclusions

Starch-based bioplastics have shown significant potential as sustainable alternatives to conventional plastics. Incorporating natural fillers, essential oils, nanoparticles, and PLA has enhanced the mechanical, thermal, and barrier properties of bioplastics, making them feasible in industrial applications. The essential oils in biocomposites showed antimicrobial activity and enhanced flexibility, whereas the starch/PLA blend had improved flexibility, reduced cost, and enhanced biodegradation. These bioplastics have been developed and explored in many industrial applications, like packaging, agriculture, textiles, the automotive industry, consumer goods, electronics, and construction, offering promising eco-friendly solutions.

However, several challenges remain, like their inherent hydrophilicity, limited thermal stability, and high production costs, but advances in material modifications, processing techniques, and the integration of biodegradable additives could help address these limitations. Moreover, while starch bioplastics present significant environmental benefits, their large-scale adoption depends on improvements in cost-effectiveness, waste management strategies, and regulatory support.

Future research should focus on optimizing formulations, scaling up production, and evaluating long-term environmental impacts to enhance commercial feasibility. Collaboration between academia, industry, and policymakers will be crucial in overcoming current challenges and promoting the widespread adoption of starch-based bioplastics in a circular bioeconomy.

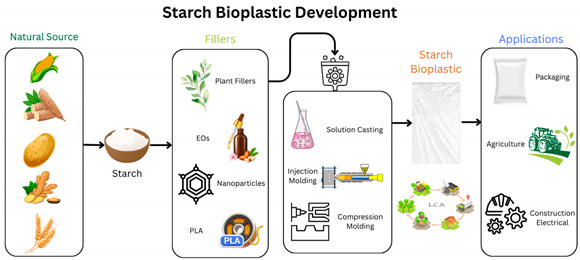



## Data Availability

No new data were created or analyzed in this study.

## Abbreviations

AESO	Acrylated epoxidized soybean oil
APTES	3-aminopropyl trimethoxy silane
BHET	Bis(2-hydroxyethyl) Terephthalate
BU	Betaine:urea
CCNC	Cellulose nanocrystals from corn husk
CCU	Choline chloride:urea
EAB	Elongation at break
EO	Essential oil
LBMD	Locust bean milling dust
LCA	Life cycle assessment
LDPE	Low density polyethylene
ODI	n-octadecyl isocyanate
OPP	Olive pit powder
PCL	Poly(ε-caprolactone)
PEO	Peppermint essential oil
PLA	Polylactic acid
PVA	Polyvinyl alcohol
ROEO	Rosmarinus officinalis essential oil
TS	Tensile strength
ZBO	Zanthoxylum bungeanum essential oil
